# Thrombolysis in central retinal artery occlusion: a retrospective observational study

**DOI:** 10.1007/s00415-022-11439-7

**Published:** 2022-10-28

**Authors:** Florian Philipp Raber, Florian Vincent Gmeiner, Jens Dreyhaupt, Armin Wolf, Albert Christian Ludolph, Jens Ulrich Werner, Jan Kassubek, Katharina Althaus

**Affiliations:** 1 Department of Ophthalmology, University Hospital Ulm, 89075 Ulm, Germany; 2 Department of Ophthalmology, ViDia Christliche Kliniken, 76135 Karlsruhe, Germany; 3 Department of Neurology, University Hospital Ulm, 89081 Ulm, Germany; 4 Institute for Epidemiology and Medical Biometry, Ulm University, 89075 Ulm, Germany

**Keywords:** Central retinal artery occlusion, Thrombolysis, Ischemic stroke, Fibrinolysis, rtPA, Magnetic resonance imaging

## Abstract

**Background:**

There is no evidence-based therapy for non-arteritic central retinal artery occlusion (NA-CRAO). Intravenous thrombolysis (IVT) with alteplase in a time window < 4.5 h may lead to a favorable outcome. Purpose of this study was to investigate the feasibility, efficacy and safety of IVT in patients classified as functionally blind.

**Methods:**

We conducted a retrospective observational study of NA-CRAO-patients. All patients underwent an ophthalmological and neurological examination including cerebral magnetic resonance imaging (MRI) for assessment of additional stroke lesions. Patients were treated either conservatively or with IVT within 4.5 h. Visual acuity (VA) was evaluated in logMAR and a categorical analysis was performed.

**Results:**

Thirty-seven patients were included in the study, 21 patients in the conservative treatment group (CTG) and 16 patients in the IVT group. The median logMAR visual acuity at admission and discharge was similar in both groups. The medium symptom to treatment time in the IVT group was 158.0 min. 3 patients (19%) of the IVT group showed a favorable outcome, all CTG patients remained at the level of functional blindness. No serious adverse events were observed after IVT. MRI showed additional acute stroke in over one-third of the patients (*n* = 14).

**Conclusions:**

Early intravenous thrombolysis therapy according to the current stroke protocol n a time window up to 4.5 h after the onset of symptoms was feasible and might be a potential treatment option for NA-CRAO. Patients with NA-CRAO are at very high risk of ischemic stroke and MRI should be done in all patients for optimized treatment and secondary stroke prevention. A prospective randomized study is required.

## Introduction

Central retinal artery occlusion (CRAO) leads to a severe and irreversible vision loss. It can be caused due to an inflammation of the artery in the context of systemic vasculitis (arteritic-CRAO, A-CRAO) or caused non-arteritic (NA-CRAO) with an etiology very similar to cerebral stroke.

In case of a NA-CRAO treatment options described include lowering of intraocular pressure, bulbar massage, isovolumic hemodilution, hyperbaric oxygen therapy, administration of antiplatelet agents and anticoagulants, surgical procedures such as paracentesis, Nd:YAG laser embolectomy and pars plana vitrectomy, and selective intra-arterial or intravenous fibrinolysis [1, 2]. Despite these numerous therapeutic options, there is no standard therapy for NA-CRAO whose efficacy could be proven in a prospective, randomized trial [2, 3]. One of the main issues is the low ischemia tolerance of the retina [4]. Experiments in monkeys have shown that a complete CRAO for less than 100 min produced no apparent damage, a CRAO of less than 240 min produced a variable degree of damage and CRAO for 240 min or more leads to irreversible damage to the neuroretina and the optic disk [5]. These data on ischemic tolerance of the retina can presumably be transferred to humans and explain why many treatment approaches simply come too late. Although there may be a spontaneous improvement in both visual acuity and visual fields within the first days in untreated patients, the risk of persistent visual impairment is very high [6]. Therefore, rapid, acute reperfusion treatment, such as in ischemic cerebral stroke, could be essential. In cases of stroke, intravenous thrombolysis (IVT) with recombinant tissue-plasminogen activator (rtPA) is the best approved therapy in a 4.5 h time window. In NA-CRAO, two prospective, randomized clinical trials on intra-arterial [7] and IVT [8] could not prove efficacy, due to the presumably too long interval until treatment [4, 9]. Recently, promising data have been published with IVT applied in a shorter time window [10–15], based on the well-studied 4.5 h time window in ischemic stroke. However, the available data are still limited and a comparison of the results is difficult due to different inclusion criteria (e.g., visual acuity at presentation, time delay of therapy and type of occlusion) and the use of different drugs that are no longer used today (e.g., streptokinase or urokinase). In this study, we investigated the feasibility, effectiveness, and safety of IVT in the treatment of acute NA-CRAO focused on patients classified as functionally blind (WHO) [16], using the established protocols for acute ischemic stroke in a 4.5-h time window with weight-adapted rtPA administration over one hour and a conservative treatment group who received conservative treatment between 4.5 and 24 h after onset of symptoms.

## Methods

We conducted a retrospective observational study of patients with NA-CRAO which were treated between 2010 and 2020 at our clinics (University of Ulm/Germany). The study was approved by the review board of the University of Ulm (reference number 36/20).

At first the patients were examined by an ophthalmologist who diagnosed the CRAO. Second, the patients were referred to the neurology department and a standard stroke workup including laboratory tests and cerebral magnetic resonance imaging (MRI) was performed [17]. A-CRAO was ruled out clinically and by laboratory diagnostics (erythrocyte sedimentation rate and C-reactive protein). Patients meeting the therapeutic window of acute ischemic stroke (less than 4.5 h) received IVT (*n* = 16) after excluding contraindications [18] and obtaining informed consent. IVT with rtPA (alteplase, Actilyse, Boehringer Ingelheim, Ingelheim, Germany) was then applied with 1 mg/kg, 10% administered as a bolus and the remainder infused over 1 h, according to the common established stroke protocol [19]. All patients were admitted to the stroke unit of the department of neurology. Patients with a symptom-to-door time > 4.5 and < 24 h were grouped in the conservative treatment group (CTG) (*n* = 21) to assess risk factors, complications and frequency of stroke.

The inclusion and exclusion criteria are presented in Table 1. In both groups, patients received “minimal” alternative treatment methods like bulbar massage and pressure lowering medications (i.e., local or systemic carbonic anhydrase inhibitors or timolol eyedrops) according to the attending ophthalmologist. The frequencies of treatment are given in Table 2.

**Table 1:** Inclusion and exclusion criteria to the study: NA-CRAO = Non arteriitic – Central retinal artery occlusion, A-CRAO – Arteriitic central retinal artery occlusion, BRAO = Branch retinal artery occlusion

Inclusion criteria	Exclusion criteria
Proven NA-CRAO Impaired visual acuity (≥ logMAR 1.3 respectively ≤ 6/120 Snellen) and normal vision before incident Arrival < 24 h after symptom onset to our study center	Other retinal artery occlusion: NA-CRAO with cilioretinal artery, A-CRAO, BRAO Other ophthalmological disease: advanced glaucoma, maculopathies, retinal vein occlusions, combined retinal arteriovenous occlusions, amblyopia, proliferative retinal diseases, previous retinal laser treatment, ophthalmologic surgery other than uncomplicated cataract surgery

**Table 2:** **Patient characteristics:** IVT = intravenous thrombolysis; IQR = interquartile rang,

	IVT-Group (0–4.5 h)	CTG (4.5–24 h)
**Patient characteristics**		
Number of Patients	16	21
Age, years ± SD	68.7 ± 14	71.8 ± 10.2
Sex, female number (%)	7 (44%)	6 (29%)
affected Eye, right number (%)	8 (50%)	12 (57%)
alternative Treatment: Bulbar massage, number (%)	4 (29%)	12 (57%)
alternative Treatment: IOP lowering medication, number (%)	6 (40%)	14 (70%)
presenting isual acuity: logMAR median (IQR)	2.30 (IQR: 2.10–2.30)	2.30 (IQR: 1.90–2.70)
final visual acuity: logMAR median (IQR)	2.10 (IQR: 1.45–2.30)	2.30 (IQR: 1.60–2.30)
symptom to treatment / neurology time in Minutes: median (IQR)	158.0 (IQR: 125.0–206.5)	480.0 (IQR: 320.0–800.0)
NIHSS at admission median (IQR)	0.5 (IQR: 0.0–1.0)	0.5 (IQR: 0.0–1.0)
Barthel Score at admission median (IQR)	100.0 (IQR: 92.5–100.0)	100.0 (IQR: 100.0–100.0)
**Systemic risk factors**		
Arterial Hypertension, number (%)	12 (75%)	18 (86%)
Atrial Fibrillation, number (%)	3 (19%)	3 (14%)
Diabetes mellitus, number (%)	4 (25%)	6 (29%)
Active Smoking, number (%)	4 (27%)	9 (45%)
History of stroke, number (%)	3 (19%)	3 (14%)
Hyperlipoproteinemia, number (%)	5 (31%)	6 (29%)
**Ocular risk factors**		
Refraction in affected eye, diopters: median (IQR)	0.188 (IQR: -0.750–1.000)	0.375 (IQR: -1.125–1.375)
Tension affected eye in mmHg: median (IQR)	16.0 (IQR: 14.0–18.0)	16.0 (IQR: 13.0–17.0)
Cataract surgery prior to incident: number %	2 (12%)	4 (22%)
**Suspected etiology of CRAO (TOAST)** [23]		
Macroangiopathic, number (%)	14	14
Cardial embolism, number (%)	0	4
Microangiopathic, number (%)	1	1
Unclear and other causes, number (%)	1	2

As clinical signs can be missing in the early CRAO, only patients with acute and painless severe vision loss (≥ logMAR 1.3 or Snellen ≤ 6/120) were included with the diagnosis of CRAO confirmed by an experienced ophthalmologist. This approach reduces the risk of including patients with other, more benign, differential diagnosis to a minimum (e.g., incomplete or transient retinal artery occlusion, branch retinal artery occlusion, optic neuritis, anterior ischemic optic neuropathy, etc.).

### Ophthalmological examination

All patients were assessed by an experienced ophthalmologist at admission and before discharge. All patients underwent an ophthalmologic examination with best corrected visual acuity (BCVA), relative afferent pupillary defect, intraocular pressure, slitlamp examination and indirect ophthalmoscopy. BCVA was measured with ETDRS charts and low vison categories were translated to logMAR according to Bach et al. [20]. Since major changes in the logMAR visual acuity at very low levels of vision may have little clinical relevance [14], a categorical analysis was carried out in visual acuity (VA) categories according to the WHO visual levels [16]: moderate visual impairment (VA: > 6/60 and ≤ 6/18), severe visual impairment (VA > 6/120 and ≤ 6/60) and blindness (VA: < 6/120). A favorable visual outcome was defined as an increase in visual acuity to ≥ 6/60 or ≤ 1.0 logMAR.

To avoid a further time delay and an increased drop-out rate to effective treatment, fluorescein angiography and other imaging procedures were not done at the first emergency visit [21].

### Neurological examination

After initial stroke assessment with a neurological examination, lab tests, and cerebral MRI according to the local stroke concept [17], all patients underwent stroke unit monitoring for at least 24 h. MRI follow-up examination was carried out in all IVT patients on the day after the IVT treatment and examined for the presence of intracerebral hemorrhage (ICH) as well as for acute and post-ischemic lesions. All patients underwent an extensive stroke workup including neurovascular ultrasound, 24-h electrocardiographic monitoring, echocardiography, and laboratory diagnostics. The NIHSS score [22] was assessed daily until discharge to quantify the impairment caused by a stroke. The suspected underlying cause was classified based on the common TOAST-classification [23].

### Statistical analysis

Visual acuity was converted to logMAR for statistical evaluation. Continuous variables were described as mean and standard deviation or median and inter quartile range (IQR) and minimum/maximum as appropriate. The Wilcoxon rank sum test was used for group comparisons of continuous data. Categorical variables were presented as absolute and relative frequencies. The chi^2^ test or Fisher’s exact test was used for comparison of categorical values as appropriate.

A two-sided *p* value of less than 0.05 was considered statistically significant. Because of the explorative nature of this study, all results from statistical tests have to be interpreted as hypothesis-generating. An adjustment for multiple testing was not made. Statistical analyses were conducted with SAS, version 9.4 (SAS Institute Inc, Cary NC, USA).

### Data availability

Any data not published within the article will be shared by the qualified investigators on reasonable request.

## Results

### Patient characteristics

Thirty-seven consecutive patients with NA-CRAO were included to the study. Sixteen Patients received IVT within a time window of 4.5 h (median symptom to treatment time = 180.0 (IQR: 150.0–250.0) minutes) according to the established stroke protocol, while 21 patients arriving between 4.5 and 24 h received conservative treatment (median symptom to neurology time = 480.0 (IQR: 320.0–800.0) minutes). The patient characteristics are given in Table 2.

### Visual outcomes after IVT

Visual acuity at admission was logMAR 2.30 (IQR: 2.10–2.30) in the IVT group (*n* = 16) and logMAR 2.30 (IQR: 1.90–2.70) in the CTG group (*n* = 21) which is corresponding to perception of hand movement (HM) in both groups (*p* = 0.38). The final VA was logMAR 2.10 (IQR: 1.45–2.30) (corresponding to counting fingers (CF)) in the IVT group and logMAR 2.30 (IQR: 1.60–2.30) (perception of HM) in the CTG group. Medium symptom to treatment time in the IVT group was 158.0 (IQR: 125.0–206.5) minutes.

Categorical analysis showed a favorable outcome in 3 patients (19%) of the IVT group: Patient I: logMAR 2.3 to 1.0 (equivalent to improvement from hand movement to 6/60); Patient II: logMAR 1.9 to 0.22 (equivalent to improvement from counting fingers to 6/9) and Patient III: logMAR 2.3 to 0 (equivalent to improvement from hand movement to 6/6). In the CTG group, all patients remained at the level of functional blindness (*p* = 0.07, Fisher´s exact test). The visual outcomes are shown in Table 3.

**Table 3:** Categorical presentation of the results according to the WHO visual levels. (VA = visual acuity; col pct = column percentage)

	IVT-Group (0–4.5 h) *N* = 16 Patients	CTG (4.5–24 h) *N* = 21 Patients
Patients n by category at admission (col pct)		
**Blindness** (VA ≤ 6/120)	16 (100%)	21 (100%)
**Patients n by category at discharge (col pct)**		
**Blindness** (VA ≤ 6/120)	13 (81%)	21 (100%)
**Severe visual impairment** (VA > 6/120 and ≤ 6/60)	1 (6%)	0
**Moderate visual impairment** (VA > 6/60 and ≤ 6/18)	0	0
**Reading ability and better** (VA > 6/18)	2 (13%)	0

### Cerebral MRI imaging findings

Of 37 patients, 37% (*N* = 14) had an additional ischemic stroke, which was in most cases clinically silent (21%, *N* = 8). Post stroke defects could be seen in 43% (*N* = 16), although only 16% (*N* = 6) had a history of stroke.

### Safety

No intracranial or intraocular hemorrhage and no other serious adverse event was observed.

## Discussion

After disappointing results from prospective randomized studies with intra-arterial and intravenous thrombolysis [7, 8] in the 20, respectively 24, hour time window, promising data for treatment within a shorter time window have recently been published. This study shows that early intravenous thrombolysis therapy according to the current stroke protocol [19] in a time window up to 4.5 h after the onset of symptoms was feasible and might be a potential treatment option for NA-CRAO. Two patients of the lysis group achieved at least reading ability and better whereas none of the conservative treatment group patients did.

In their meta-analysis, Schrag et al. [11] reported results of patients treated in the 4.5 h time window (antifibrinolytic agent: streptokinase, urokinase and rtPA). The rate of visual recovery, defined as the percentage of patients with a visual acuity increase from ≤ 6/60 to ≥ 6/30, was 50.0% (17 of 34 patients) in patients treated within the 4.5-h time window. This compared favorably to the spontaneous course in the untreated patients with a recovery rate of 17.7% (70 out of 396 patients).

This meta-analysis was updated in 2020 by Mac Grory et al. [15] to include more recent data and further studies [8, 12–14]. For the 67 patients treated with the more recent fibrinolytic agent rtPA in the 4.5-h time window, a recovery rate of 37.3% was reported.

Although the studies are not directly comparable due to different inclusion criteria, in particular presenting VA ≤ 6/60 chosen in the meta-analysis and 6/120 in our study, the rate of visual recovery according to Schrag´s definition [11] is lower in our study. Only 12.5% of patients recovered according to their definition of visual recovery (2 patients improved from CF/HM to 6/9 resp. 6/6). One possible reason for the lower recovery rate in our cohort was the stricter inclusion criteria with a visual acuity ≤ 6/120 which represents functional blindness according to the WHO´s definition and could have led to the selection of more severe cases. The more stringent visual acuity inclusion criteria tend to exclude other benign differential diagnoses (e.g., anterior ischemic optic neuropathy, optic neuritis, transient retinal arterial occlusion).

Among the studies of Mac Grory et al. [15] (*n* = 16) and Schultheiss et al. [14] (*n* = 20), we describe one of the largest independent cohorts published to date, that contains patients treated with rtPA in a 4.5 h time window.

In the observational cohort study of Mac Grory et al., including patients with a visual acuity ≤ 6/60, reported a recovery rate of 43.8% (*n* = 16 Patients, improvement VA from ≤ 6/60 to ≥ 6/30) for patients treated with rtPA in a time window of 4.5 h. 31.2% achieved visual acuity ≥ 6/19 and thus reading ability and better. Interestingly a visual recovery of the untreated group (*n* = 43) was achieved in 11.6% of cases in a 4 h’ time [15].

Schultheiss et al. [14], showed in a prospective interventional case-series that 20 patients who were treated with an IVT (rtPA) in < 4.5 h with severe vision loss (≤ 6/120 = functional blindness) regained reading ability (VA ≥ 6/20) in 25% of the cases compared with 5% of the cases of the conservative treatment arm of the EAGLE Study [7] at day 30. The number of patients in our cohort who benefited from fibrinolysis appears lower. The comparison of the "symptom to treatment time" in our cohort (162.5 ± 57 min (mean ± SD) with that of Schultheiss et al. (183.5 ± 62 min (mean ± SD) [14]) shows a slightly faster treatment in our study population. Patients with favorable outcomes were treated early (patient I: HM to 6/60, symptom to lysis time 270 min; patient II: CF to 6/9, symptom to lysis time 180 min and patient III: HM to 6/6, symptom to lysis time 74 min). Despite the data on ischemia tolerance from primate experiments [4, 5], an important question for future studies will be up to which time window patients could benefit from this possible therapy’.

The number needed to treat in ischemic stroke for a favorable outcome, means a functionally independent patient is 14 within 4.5 h of treatment onset [24], and is apparently similar to our results of IVT in NA-CRAO.

The main argument against the use of intravenous fibrinolysis in acute CRAO is concern about the risk of hemorrhagic complications, particularly intracerebral or intraocular bleeding. As shown in many studies on ischemic stroke, thrombolysis < 4.5 h after symptom onset is safe [17, 25, 26]. This corresponded to our safety data since we did not observe any bleeding complications in our sample, even though the total number (*n* = 16) of thrombolysed patients in our cohort was too limited to draw final conclusions.

The rate of patients with acute cerebral ischemia on MRI with central artery occlusions was 37% in our study. These data are consistent with a recent meta-analysis by Fallico et al. [27] where the rate of cerebral ischemia was 30% for CRAO and 25% for BRAO. Patients with CRAO have a very high risk of additional acute strokes. Although these mostly minor cerebral infarctions did not cause apparent neurologic deficits in the majority of our patients, these incidental findings are extremely important not only for the differential ethological considerations depending the underlying cause of the acute ischemic event but especially for secondary prevention of future strokes [28].

In this work we present one of the largest, well-characterized cohorts of patients treated with rtPA with acute severe vision loss. Extensive ophthalmological and neurological evaluation of the patients was performed.

Our study is limited by its retrospective character, however, to maintain a short door to treatment time a strict scheme of standard operating procedure was applied resembling to a prospective treatment protocol.

Additionally, our study, is not a comparative study, and the comparison of visual acuity between the treatment group (0–4.5 h) and the conservative treatment group (4.5–24 h), in terms of retinal ischemia tolerance is not possible. However, evaluating patients within a time window up to 24 h is important when evaluating other parameters (complication rates, occurrence of cerebral ischemia, development of visual acuity). Data on visual acuity in untreated patients at 0–4.5 h have already been published in various studies [7, 11, 14, 15]. In our cohort, both groups received “minimal” additional treatment by means of bulbar massage and, if necessary, pressure-reducing therapy according to the assessment of the treating ophthalmologist. Due to time constraints, the patients from the CTG group received these treatment methods more frequently, although there is no scientific evidence of their effectiveness. Other more aggressive therapies (e.g., vitrectomy/laser or paracentesis) were not used. For future studies, we recommend to perform an OCT prior to lysis therapy, as it enables to confirm the diagnosis and, based on new data [29], may also provide a prognostic assessment. Overall, the authors advocate the implementation of a prospective randomized placebo-controlled multicenter study on the effectiveness of systemic lysis therapy for retinal central artery occlusion, although the realization will be challenging given the required multidisciplinary efforts to this rare disease in a short time window.

## Conclusions

This single-centre retrospective observational study of functional blind NA-CRAO-patients shows that intravenous fibrinolysis therapy within a multidisciplinary approach was feasible. Of the 16 patients, 3 (19%) had improved vision after thrombolysis in an early time window of 4.5 h after symptom onset. No systemic complications were observed. Patients with CRAO are at very high risk of additional ischemic strokes and MRI should be done in all patients for optimized treatment and secondary stroke prevention. A prospective, randomized, placebo-controlled clinical trial is required to prove dosage, optimal time window, and efficacy of intravenous fibrinolysis in an early time window for NA-CRAO.

## Data Availability

Any data not published within the article will be shared from the qualified investigators on reasonable request.
